# Opening Wedge High Tibial Osteotomy with Combined Use of Patient-Specific 3D-Printed Plates and Taylor Spatial Frame for the Treatment of Knee Osteoarthritis

**DOI:** 10.1155/2021/8609921

**Published:** 2021-12-01

**Authors:** Desheng Duan, Yang Cao, Renzeng Li, Guohui Wang, Yongfei Zhang, Kui Xiang, Yanli Hu, Yiqun Li, Peng Peng, Pan Zhang, Xianzhe Liu

**Affiliations:** ^1^Second Department of Orthopaedics, Third People's Hospital of Anyang City, Anyang 455000, Henan Province, China; ^2^Zhengzhou University of Light Industry, Zhengzhou, Henan Province, China; ^3^Department of Plastic Surgery, The Central Hospital of Wuhan, Affiliated Tongji Medical College, Huazhong University of Science and Technology, Wuhan 430014, Hubei Province, China; ^4^Department of Orthopedics, Union Hospital, Tongji Medical College, Huazhong University of Science and Technology, Wuhan, China

## Abstract

**Background:**

High tibial osteotomy (HTO) is used to treat medial degeneration of the osteoarthritis (OA) knee. However, shortcomings still exist in the current procedure, like unprecise creation, inability to correct knee rotation, and internal fixed failure. Here, we reported a novel procedure: patient-specific 3D-printed plates for opening wedge high tibial osteotomy (OWHTO) combined with Taylor spatial frame (TSF). The detailed technique was described, and the clinical outcomes were evaluated.

**Methods:**

We prospectively evaluate outcomes of patient-specific 3D-printed plates for OWHTO with use of TSF in 25 patients with knee OA and varus alignment. Postoperative efficacy was evaluated using the HSS knee score, pain visual simulation score (VAS), and knee joint motion (ROM), and lower limb alignment was evaluated by measuring femorotibial angle (FTA) and hip-knee-ankle (HKA). *Results and Conclusion.* All patients did not experience complications such as wound infection, nerve damage, or bone amputation. 25 patients were followed up for 6–18 months. The bony union at bone amputation was achieved in 3 months after surgery, and the pain symptoms were significantly alleviated or disappeared. The VAS score was significantly reduced in 6 months after surgery compared with preoperative; the HSSS score was significantly added in 6 months after surgery compared with preoperative. The ROM of knee joint increased significantly 6 months after operation compared with that before operation, and the difference was statically significant (*P* < 0.05). The FTA and HKA after operation were significantly superior to that before operation, and the difference was statically significant (*P* < 0.01).

**Conclusions:**

Our study showed that patient-specific 3D-printed plates for HTO with the use of TSF have the advantages of small trauma, few complications, simple operation, and fast recovery in treating knee OA and varus alignment.

## 1. Introduction

The high tibial osteotomy (HTO) is a safe and effective knee-saving procedure for the treatment of early knee osteoarthritis, which relieves arthritis symptoms and prolongs the life of the knee joint. The open wedge high tibial osteotomy (OWHTO) has been widely used as one of the most common HTO techniques. But at present, OWHTO with a locked proximal humeral plate still has the shortcomings of inability to accurately control the lower limb alignment, unsuitability for dealing with larger varus deformity of tibia, inability to correct knee rotation or compound malformation, bone nonunion at amputation bone, internal fixed failure, and other shortcomings [1]. To overcome the above deficiencies, we tried to use TSF outrigger as outer fixed support of OWHTO in treatment of knee osteoarthritis.

## 2. Materials and Methods

### 2.1. Inclusion and Exclusion Criteria

Inclusion criteria: in line with the diagnosis of knee osteoarthritis and for the inner chamber type, less than (including) 65 years old, knee joint with general stability and normal muscle strength, overall-length X-rays at weight-bearing of double lower limb show normal outside gap of knee joint, but narrow inner gap, and patients who cooperate with treatment and completion of follow-up visits.

Exclusion criteria: knee osteoarthritis with internal and external chamber narrowness, explicitly diagnosed with rheumatoid knee arthritis or infection around the knee joint, posttraumatic knee arthritis, combined with severe underlying diseases intolerable to surgery, and those who have not been treated as directed by the doctor and complete follow-up.

### 2.2. Patients' Information

According to the above criteria of inclusion and exclusion, 25 patients voluntarily enrolled, as given in Table 1.

### 2.3. Preoperative Planning

Movement trajectory and target alignment of osteotomy were designed by computer, the 3D-printed guide plate of osteotomy was designed, and then, the bone depth was calculated through the guide plate; PLA (polylactic acid) which met the requirements of FDA was used to print the 1 : 1 model and osteotomy guide plate of the upper tibia of the patients. The 3D-printed guide plate was manufactured by a commercial 3D printer (Formlabs, Cambridge, MA) using biocompatible resin (Dental SG, EN-ISO 10993–1:2009/AC:2010, USP Class VI). After printing, they were sterilized by low-temperature plasma. Before clinical use, moist heat sterilization was adopted, and no visible deformation of the 3D-printed guide plate profile was allowed to affect the accuracy.

### 2.4. Surgical Procedure

**Figure 1:**
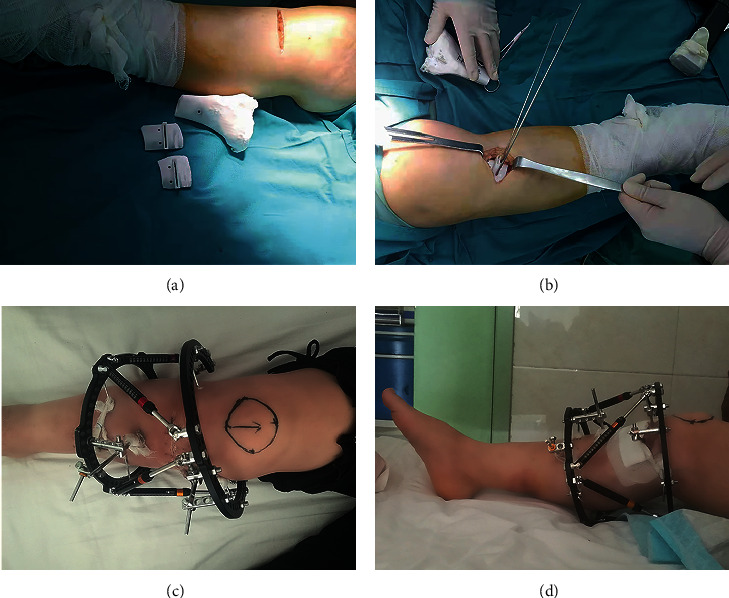
(a-b) Intraoperative photograph with the 3D-printed plates guide. (c-d) Postoperative photographs of TSF.

Patients were maintained at supine position for epidural anesthesia. An incision of about 6 cm toward the upper rear should be made from the lower part of the tibial tuberosity (front edge of goose palm) to the rear inner corner of medial tibial plateau, the goose palm tendon was retracted to the far end to reveal the upper edge of the superficial parts of collateral ligament, and then insert the periosteum elevator under the ligament and lift it from the tibia(Figure 1(a)). Use a scalpel to separate the long fibers of the superficial part of the ligament from the tibia until the shin bone is exposed and insert into the Hohmann hook after the tibia. Placement of the guide plate, 2 Koch needle fixed, see the guide plate position of osteotomy as presurgery planning, and then conduct “one-size-fits-all” osteotomy through the guide plate in accordance with the depth of preoperative measurement, that is, a osteotomy line (2-size-fits-all is used for AO standard to cut 2 faces), the osteotomy design is from the middle and lower 1/3 of tibia nodule to lower 1/3 of the fibula head to avoid postoperative tibia upward movement and the affection to the trajectory of the tibia, and second, the line is located in the cancellous bone of metaphysis, which is easier for the end of the osteotomy end to heal (Figure 1(b)). When the position of osteotomy is satisfactory through fluoroscopy, withdraw the swing saw and guide plate, repeatedly rinse the incision until the bleeding is completely stopped, and close the incision layer by layer. Then, penetrate into 2 K-wires of 2.0 mm and 1 threaded bone needle on joint surface at the near end of the osteotomy plane parallel to the tibia platform, penetrate into the other 2 K-wires of 2.0 mm and 2 threaded bone needles at about 12 cm of the far end of the osteotomy plane, and then install TSF with full independent intellectual property rights and computer patent prescription (Tianjin Xinzhong) and tighten all fixed screws, with 6 rods all connected at 15 mm. If the position of the K-wires and threaded bone needle is satisfactory, bandaging incision and needle passage with sterile dressing, the surgery is completed. The TSF would be adjusted according to the “receipt,” planed before the surgery (Figures 1(c) and 1(d)). The aim of adjustment is correcting the varus deformity of tibia and lower limb alignment. The frame would be removed after 3 months after the operation normally.

### 2.5. Postoperative Evaluation

**Figure 2:**
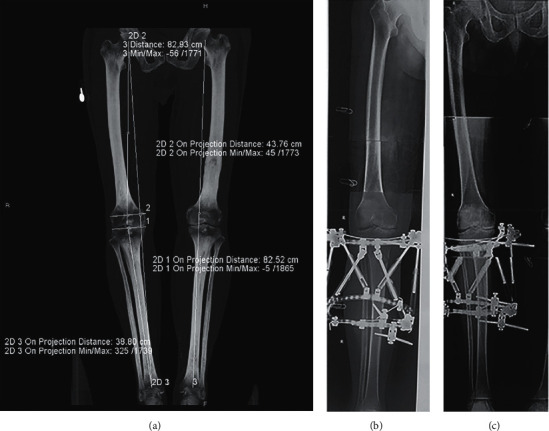
(a) 53-year-old male suffered with knee osteoarthritis combined with knee varus (left: preoperative X-ray shows hyperosteogeny at the edge of the right knee joint, tibial eminence, upper and lower margin of the patella, hardening of joint surface, and the narrowing of joint spaces inside the knee). TSF-assisted OWHTO surgery was conducted on the right knee (b). One month after surgery, the full-length X-ray of the double lower limb shows that correction of the right lower limb assignment is fair (c).

**Figure 3:**
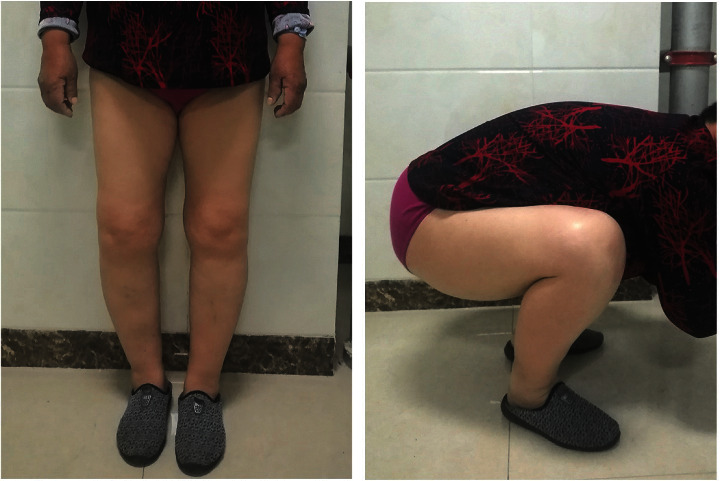
Postoperative photographs of a patient in standing and squatting positions three months after surgery.

Hospital for special surgery knee scoring system (HSS) and visual analogue scale (VAS) were used for clinical evaluation. The weight-bearing radiographs of the full legs were used for measuring the femur angle (FTA) and hip-knee-ankle angle (HKA) before and after 6 weeks of surgeries [2]. A typical case for postoperative evaluation is shown in Figures 2 and 3.

### 2.6. Statistical Analysis

All statistical tests were performed in Software Package for Social Sciences (SPSS) Statistics version 25.0.

## 3. Results

25 patients were followed up for 6–18 months. The rate of bone union is 100% in three months. VAS score significantly decreased 6 months after surgery compared with that before surgeries. HSS scores and ROM scores significantly increased 6 months after surgeries (Table 2).

## 4. Discussion

Since 1960s, HTO surgery has continuously developed. Opening wedge high tibial osteotomy (OWHTO) with a locked proximal humeral plate has been considered to be a relatively simple and safety treatment for knee OA with genu varum [3]. However, OWHTO relies on surgeon's experiences of controlling the lower limb assignment and determining the angle and size of wedge-shaped osteotomy. Moreover, the conventional surgery loses the opportunity to adjust again regardless of the insufficient or excessive correction assignment [4]. For severe tibia deformity, like existing knee-turning, rotation, or compound malformation, the rate of failure is relatively high [5].

It was reported that the success of HTO requires at least three important factors: appropriate patient selection, safe and accurate surgical techniques, and reliable internal fixation [6]. Thus, these factors lead to a steep learning curve [7]. Previous literatures suggested that the navigation system allowed a significantly better tibial slope control and Lysholm scores when patients underwent HTO [8]. Patients operated with the navigation system had significantly better Lysholm scores. Munier et al. showed that the use of patient-specific cutting guides in HTO procedures helps to achieve optimal correction in a safe and reliable manner [9]. The correction was achieved through the alignment of the predrilled holes and the plate holes. Compared with the current study, the predrilled method allowed a smaller volume of PSI when a short plate is used; thus, a minimum incision can be attained. To achieve a good surgical effect requires continuous study and reflection. The application of TSF can solve the shortcomings of traditional HTO surgery [10]. It improves the accuracy of osteotomy through 3D preoperative planning. Slow distraction osteogenesis can avoid overcorrecting. As the TSF is a relatively mature technology, the learning curve is relatively shallow. Through the computer's precise calculation, a “correction prescription” is developed individually for each patient through the slow distraction osteogenesis, achieving the purpose of correcting in three dimensions and six degrees.

Although open reduction and internal fixation is usually better tolerated by patients and requires less frequent follow-up and radiographic evaluation, the deformity correction cannot be changed postoperatively. The application of TSF on the correction of the rotation of the limbs and bone joints on the plane and deformity shortening is a very mature technology [11]. HTO with TSF was presented as a viable treatment option in active patients with early medial compartment OA [12]. After the computer's precise calculation, a “correction prescription” is developed individually for each patient through the slow distraction osteogenesis to achieve the purpose of while correcting while building up bones in three dimensions and six degrees.

After OWHTO in the operation, wedge-shaped prognosis is not conducted, the TSF is used instead of locking the bone plate for fixing, the operation is very simple, after surgery “correction prescription” is produced individually according to the accurate calculation by the computer for accurate correction, and the rotation or compound malformation on each plane can be corrected, meanwhile the osteotomy end is slowly stretched into the bone, which greatly reduced the bone amputation or disunion and other complications [13]. The impact of surgery on the patient's daily life is lowered to a maximum extent. The patients in this study all achieved good short-term clinical efficacy. Yunhe et al. compared the clinical outcomes of the MOWHTO using 3D-printed patient-specific instrumentation with that of conventional surgical techniques. The results showed MOWHTO using 3D-printed patient-specific instrumentation is safe and feasible, with superior accuracy than the conventional technique. In their study, the postoperative mFTA was over 180 degrees, while our results showed 170.33 ± 3.08 degrees at the 6-month follow-up [14].

In this study, none of the patients had incision infection. The dressing was changed every other day, and iodophor gauze was applied until TSF was removed. In this study, there was not a case of superficial infection at the nail path. Compared with HTO surgery, the low infection rate of incision is also one of the advantages of the application of TSF. Theoretically, the stability of TSF is not as good as that of the locking plate fixation [15]. However, in this study, there was not a case of nonunion of the fracture, indicating that the stability of the external fixation is sufficient, and the external frame is fixed below the knee joint of the patient. The impact of the inconvenience of the patient's daily life is limited to minimum, so the patient can recover early. The early rehabilitation activities can stimulate the bone healing at the osteotomy, which in turn enhances the stability of the fracture [16].

## 5. Conclusions

In summary, the results of this study show that TSF-assisted OWHTO is a precise and effective technical means to treat knee OA with genu varum, which has the advantages of simple operation, small trauma, few complications, and fast recovery, and has a certain clinical application value. However, the sample size in this study is small, and the follow-up time is short. Moreover, this study did not set up a control group.

Thus, a larger cohort and randomized controlled trial with long-tern follow-up is needed in the future.

## Figures and Tables

**Table 1:** Patients demographics.

Age, years	55.8 ± 8.8
Sex (male : female)	12 : 13
Right : left	15 : 10
BMI	25.9 ± 3.15

**Table 2:** Clinical evaluation before and after surgery ($\bar{x}\pm s$, *n* = 25).

Time point	VAS	HSS	RO (°)	FTA(°)	HKA (°)
Preoperative	6.01 ± 1.07	51.27 ± 11.35	80.55 ± 10.27	176.45 ± 1.19	165.42 ± 3.76
6 months	2.44 ± 0.98	79.31 ± 12.06	102.38 ± 16.51	170.33 ± 3.08	185.57 ± 4.35
*P* value	<0.001	<0.001	<0.001	<0.001	<0.001

VAS, pain visual simulation score; HSS, knee joint scoring system; ROM, mobility of knee joint; FTA, femorotibial angle; HKA, hip-knee-ankle.

## Data Availability

The datasets used and/or analyzed during the current study are available from the corresponding author upon request.
